# PIWIL2 promotes progression of non-small cell lung cancer by inducing CDK2 and Cyclin A expression

**DOI:** 10.1186/s12967-015-0666-y

**Published:** 2015-09-15

**Authors:** Xiaohan Qu, Jinlu Liu, Xinwen Zhong, Xi Li, Qigang Zhang

**Affiliations:** The First Affiliated Hospital, China Medical University, NO. 155, Nanjing North Street, Heping District, Shenyang, 110001 Liaoning China; The Forth Affiliated Hospital, China Medical University, Shenyang, China

**Keywords:** PIWIL2, Non-small cell lung cancer, Cyclin A, CDK2, Prognosis

## Abstract

**Background:**

PIWI proteins have important roles in tumorigenesis due to their interaction with piRNAs. Recent studies suggest that PIWI proteins affect prognosis of various cancers.

**Methods:**

In the present study, PIWI genes expression was assayed in non-small cell lung cancer (NSCLC). To determine the effects of PIWIL2 on NSCLC cells, overexpression and interference assays were performed using the A549 and H460 cell lines. The tumor formation model was performed to demonstrate the effects of PIWIL2 on tumor formation in vivo.

**Results:**

PIWIL2 was increased both at the RNA and protein level in malignant cancer tissues compared with adjacent normal tissue. Moreover, increased PIWIL2 gene expression was negatively correlated with prognosis in NSCLC patients. Overexpression and interference of PIWIL2 promoted and depressed cell proliferation, respectively. Meanwhile, PIWIL2 interference arrested cells at the G2/M stage. In addition, we found that CDK2 and Cyclin A expression were correlated with PIWIL2 expression. Moreover, transfection of PIWIL2 promoted tumor growth in nude mice.

**Conclusion:**

Our findings shed light on the function of PIWIL2 in NSCLC and suggest potential prognostic and therapeutic value.

## Background

Non-small cell lung cancer (NSCLC) is one of the most common cancers worldwide and is associated with high mortality rates [1]. Several biomarkers have been used to detect progression have been used in diagnosis of NSCLC mortality. However, both NSCLC-specific antigens and mRNA-based biomarkers are associated with overdiagnosis and overtreatment [2]: mRNA-based biomarkers are seldom specific to NSCLC tissue and are commonly expressed by epithelial cells, including normal, hyperplastic and cancerous cells [2]; meanwhile, NSCLC-specific antigen serum levels could reflect cancer progression or NSCLC hyperplasia, infection or inflammation [3]. Thus, there is a need for improved NSCLC biomarkers. Meanwhile, the most common therapeutic strategy for NSCLC, including exairesis, chemotherapy and radiotherapy, are associated with poor prognosis and low long-term survival rates [4]. New strategies such as gene therapy and stem cell therapy have been used in clinical treatment [5, 6]. In addition, epigenetic research presents another potential cancer therapeutic avenue; understanding epigenetic changes during NSCLC progression could contribute to diagnosis, predicting prognosis and exploring new treatment strategies [7, 8].

Chromosomal instability is a common feature of cancer cells that often displays deletions, rearrangements and duplications of different DNA fragments in the genome [9]. Specific alterations in miRNA expression, methylation levels and histone modifications are all regarded as causative factors in the etiology of cancer [10, 11]. Indeed, aberrant DNA methylation has been found in transposable elements (TEs)–regions that may be associated with cancer progression [12]; erratic TEs could lead to genomic instability and affect expression levels of both oncogenes and tumor suppressor genes [13, 14]. Hyperactive TEs therefore appear to play an integral role in cancer development. PIWI proteins are key factors in controlling TE stability [15, 16]. By interacting with PIWI-interacting RNAs (piRNAs), PIWI protects the integrity and stability of the genome from silencing TEs [17]. Much progress has been made in understanding the molecular function of PIWI in several cancers; however, the role of PIWI in NSCLC remains unknown.

Although the PIWI-piRNA pathway was first identified in the testis, PIWI has subsequently been found to be highly expressed in many different types of cancer [18]. PIWI is expressed in breast cancer [19] and gastrointestinal cancer [20, 21] but not in the corresponding normal tissues. PIWI homologs are common in vertebrates; for example, in mice, three different homologs of PIWI were found [22] while in humans, PIWIL1, PIWIL2, PIWIL3 and PIWIL4 were identified [23, 24]. These homologs may have different functions depending on the type of cancer. PIWIL1 promotes cancer growth and is associated with increased mortality [20, 25]; increased levels of PIWIL2 have been reported in breast [26, 27] and cervical cancer [28]; and increased levels of PIWIL3 and PIWIL4 have been reported in colon cancer [29]. To date, PIWI gene expression has not been investigated in NSCLC; the expression patterns and function of PIWI in NSCLC therefore warrants investigation.

In the present study, we focus on the expression pattern of PIWI homologs in the context of NSCLC progression and prognosis. We investigated the function of PIWI in a NSCLC cell line, using cell transfection. The biological activities of the NSCLC cell line were confirmed after PIWI over-expression and silencing. Finally, Cyclins and Cyclin-dependent kinases (CDKs) were detected after PIWI over-expression and silencing to investigate the mechanism of PIWI on NSCLC cell progression both in vivo and in vitro. This study provided novel insights into the role of PIWI in NSCLC that suggest potential diagnostic, prognostic and therapeutic value for PIWI in NSCLC.

## Methods

### Materials

NSCLC samples were provided by the China Medical University (Shenyang, China) in accordance with the guidelines of the China Medical University of Medicine Research Ethics Committee. All patients provided informed consent. NSCLC tissues and adjacent tissues were collected from patients who underwent resection. Tumor tissues were confirmed as such by haematoxylin and eosin staining. Only those samples with more than 70 % tumor content were used for further study as tumor tissues.

### Cell culture and transfection

The human NSCLC cell line, A549 and H460 were obtained from the Shanghai Cell Bank at the Chinese Academy of Sciences. The cells were cultured in Dulbecco’s modified Eagle’s medium (DMEM) (Gibco, Gaithersburg, Maryland, USA) containing 10 % fetal bovine serum (Gibco) at 37 °C in a incubator at 5 % CO_2_.

The pcDNA3.1+ vector containing c-Myc-tagged PIWIL2 was constructed in our laboratory as previously described [30]. The c-Myc promoter was synthesized by Sangon Biotech (Shanghai, China). The PIWI fragment was amplified from human iPS cell lines with the primer set: 5′-TTC TCG AGA TGG ATC CTT TCC GAC CAT C-3′ and 5′-TTC CAT GGT CAC AGG AAG AAC AGG TTC TC-3′. The primer sets were designed to contain the full length of coding sequence. Amplified PIWI cDNA was digested with NcoI and XhoI (New England BioLabs [NEB], Hertfordshire, UK). The digested fragment was then subcloned between the NcoI site and the XhoI site of the pcDNA3.1+ vector. The construction of shRNA for PIWIL2 was previously reported [30] (PIWIL2 shRNA1: 5′-ACC GGC CUG GGU UGA ACU AAA-3′, PIWIL2 shRNA2: 5′-ACA GAA UCA AAC ACU GUG A-3′). Transfection was performed in 6-well plates with Lipofectamine 2000 (Invitrogen, Carlsbad, CA, USA) according to the manufacturers’ protocol. Cells were then replated at a density of 2 × 10^5^ cells per well and cultured for 48 h after which they were assayed by real-time PCR, western blot and immunofluorescence staining. For rescue groups, after 24 h post interference or over-expression transfection, the second over-expression transfection or interference were performed, respectively. Then the cells were analyzed at 48 h post the second transfection.

### MTT assays

To assay the cell proliferation rate, 24-well plates at a density of 2 × 10^4^ cells/well were seeded after transfection. Subsequently, the cells were analyzed by methyle thiazol tetrazolium assay (MTT assay, Sigma-Aldrich, St. Louis, MN, USA). The amount of MTT formazon product was determined by using a microplate reader and the absorbance was measured at 570 nm (Berthold, Tokyo, Japan).

### Real-time PCR

Total RNAs were extracted from tissues or cells using Trizol (Invitrogen, Carlsbad, CA, USA). The quality and quantity of total RNA was determined by agarose gel electrophoresis and by the BioPhotometer Plus (Eppendorf AG, Hamburg, Germany). For reverse transcription, 1 μg total RNA was transcribed for each sample to complementary DNA (cDNA) using M-MLV reverse transcriptase (Invitrogen) according to the manufacturer’s protocol. The primers for real-time PCR are shown in Table 1. The PCR conditions were as follows: 95 °C for 3 min, 40 cycles at 95 °C for 12 s and 55 °C for 40 s. GAPDH was used as an internal PCR control. Meanwhile, a no-template control and melt curve analysis were used to monitor contamination. The relative expression levels were calculated using the 2^−ΔΔCt^ method.

**Table 1:** Primers used in the present study

	Forward 5′–3′	Reverse 5′–3′
PIWIL1	TGTCTGTTGTCAAGTAATCGGAAGG	TTGCTGTTTGCCTAAGGTTCG
PIWIL2	TACCTTCAGCACACCGTCC	GACACTGTATTTTGACGAGGT
PIWIL3	GAGCCCAGATACAGTACAGCGTT	GGACTGCCCCACGAGGTAA
PIWIL4	TACTGTATCGGACCTGAATCA	TTCAGCCACAGCCTTCATCAG
CDK1	TGCTAAGTTCAAGTTTCGTAATGCT	AAGGACTGAGATGATTTAAGCCAAC
CDK2	ATCCGCCTGGACACTGAGACT	TGGAGGACCCGATGAGAATG
CDK4	CACAGTTCGTGAGGTGGCTTTA	TGTCCTTAGGTCCTGGTCTACATG
Cyclin B	TTGGTTTCTGCTGGGTGTAGG	CCATGTTGATCTTCGCCTTATTT
Cyclin A	GCATGTCACCGTTCCTCCTTG	GGGCATCTTCACGCTCTATTTT
Cyclin D1	TCGCTGGAGCCCGTGAA	CCGCCTCTGGCATTTTGG
GAPDH	CGCTCTCTGCTCCTCCTGTT	CCATGGTGTCTGAGCGATGT

### Western blot

The levels of PIWI proteins, CDK proteins, Cyclin proteins and GAPDH were determined by western blot. Primary rabbit polyclonal antibodies against PIWIs (PIWIL1, SAB1300682; PIWIL2, SAB2105190; PIWIL3, SAB4200150; PIWIL4, SAB2105885), CDKs (CDK1, SAB4500050; CDK2, SAB4503706; CDK4, SAB4300695), Cyclins (Cyclin B, SAB4503501; Cyclin A, SAB4503499; Cyclin D1, SAB4502603) and GAPDH (SAB2100894) were purchased from Sigma-Aldrich (St. Louis, MO, USA). Secondary antibodies were HRP-conjugated anti-rabbit IgG (Sigma-Aldrich). Both tissues and cells were homogenized using RIPA buffer (50 mM Tris pH 8, 150 mM NaCl, 1 % NP-40, 0.5 % DOC, 0.1 % SDS, 1 mM DTT, protease and phosphatase inhibitors). Extracted proteins were quantified using the BCA protein assay kit from Sangon Biotech (Shanghai, China). For each sample, 15 μg protein were electrophoresed on a 12 % sodium dodecyl sulfate polyacrylamide gel. The sample was next transferred onto polyvinylidene fluoride membranes (Millipore, Billerica, MA, USA). Proteins were blocked at room temperature for 1 h using 4 % skim milk and incubated with each antibody (for PIWIs, 1:1000; CDKs, 1:1000; Cyclins, 1:100; GAPDH, 1:2000) at 4 °C overnight. After three washes with TBST buffer (pH 7.6, 20 mM Tris–HCl, 137 mM NaCl, 0.01 % Tween-20), membranes were incubated with HRP-conjugated anti-rabbit IgG and visualized using enhanced chemiluminescence (ECL, Millipore, Billerica, MA, USA).

### Immunohistochemical staining (IHC)

Tissue samples were fixed with 10 % buffered formalin and 6-μm-thick tissue sections were cut. The sections were then incubated with monoclonal antibody (for PIWIs, 1:100; CDKs, 1:200; Cyclins, 1:200) and the same secondary antibody that was used for western blotting. Horseradish peroxidase streptavidin complex (Beyotime, Wuhan, China) was used to visualize the signals. Sections were color-developed with diaminobenzidine and stained with hematoxylin (Beyotime).

### Immunofluorescence microscopy

Protein expression was measured by immunofluorescence, using monoclonal antibodies (for CDKs, 1:100; Cyclins, 1:100) that were detected by FITC-conjugated goat anti-rabbit IgG (Sigma-Aldrich) at a dilution of 1:200. DAPI (1 μg/ml) was used to stain nuclei.

### Flow cytometry

Cell activities and apoptosis were measured by flow cytometry after over-expression and inhibition of PIWIL2 in NSCLC cells. Annexin V-FITC and propidium iodide (PI) (BioVision, Milpitas, CA, USA) staining was used according to the manufacturer’s instructions. For each sample, at least 30,000 cells were analyzed. Experiments were performed in triplicate.

### Tumor formation in a nude mouse model

Five-week-old nude mice were used to confirm the effect of PIWIL2 on tumor formation. The experiment was approved by the ethics committee of the China Medical University (Shenyang, China). Mice were randomly divided into two groups of ten (n = 5). One group was treated with A549 cells while other was treated with A549 cells containing PIWIL2 expression vectors; 30,000 A549 cells (in 1 ml DMEM) were injected every 3 days for 21 days on the dorsal side of the mouse. Tumor volumes were calculated every 3 days according to the following the formula: Volume = length × width × height × 3.14/6. At 21 days, tumor tissues were assayed by real-time PCR, western blot and IHC.

### Statistical analysis

Real-time PCR, MTT assays and apoptosis rates are presented as mean ± standard deviation (SD). One-way-ANOVA analysis was used to analyze differences among groups and results where P < 0.05 were considered significant. Survival curves were plotted according to the Kaplan–Meier method. Of the 126 patients involved in this study, 62 expressed relatively low levels of PIWIL2 (fold-change relative expression to adjacent tissue was between 2 and 4) while 64 expressed high levels of PIWIL2 (fold-change relative expression to adjacent tissue was between 5 and 7) in cancer tissues, as judged by real-time PCR (Table 2). Multivariate analysis was conducted using Cox’s proportional hazards regression model. Patients’ survival information was collected telephonically. Tumor stage was classified based on the 2011 Union for International Cancer Control (UICC) TNM classification of malignant tumors. The nuclear grading was performed according to Fuhrman’s system [31]. All data were analyzed using SPSS 17.0 (SPSS Inc., Chicago, IL, USA).

**Table 2:** Clinical characteristics of patients with liver cancer in the present study

Parameters	n	PIWIL2		χ^2^	P value
		High	Low		
Total	126	64	62	0.189	0.87
Sex					
Male	60	30	30		
Female	66	34	32		
Age (years)				1.56	0.56
≥50	67	34	33		
<50	59	30	29		
T stage				6.15	0.03
T1	75	40	35		
T2	32	18	14		
T3/4	19	6	13		
N stage				4.62	0.05
N0	96	44	52		
N+	30	20	10		
Metastasis				5.69	0.03
No (M0)	72	35	37		
Yes (M1)	54	29	25		
Recurrence				7.62	0.02
No	70	34	36		
Yes	56	30	26		
Fuhrman				11.52	0.02
1	21	11	10		
2	64	30	34		
3	22	11	11		
4	19	12	7		

## Results

### PIWIL2 expression is elevated in NSCLC tissues

**Fig. 1:**
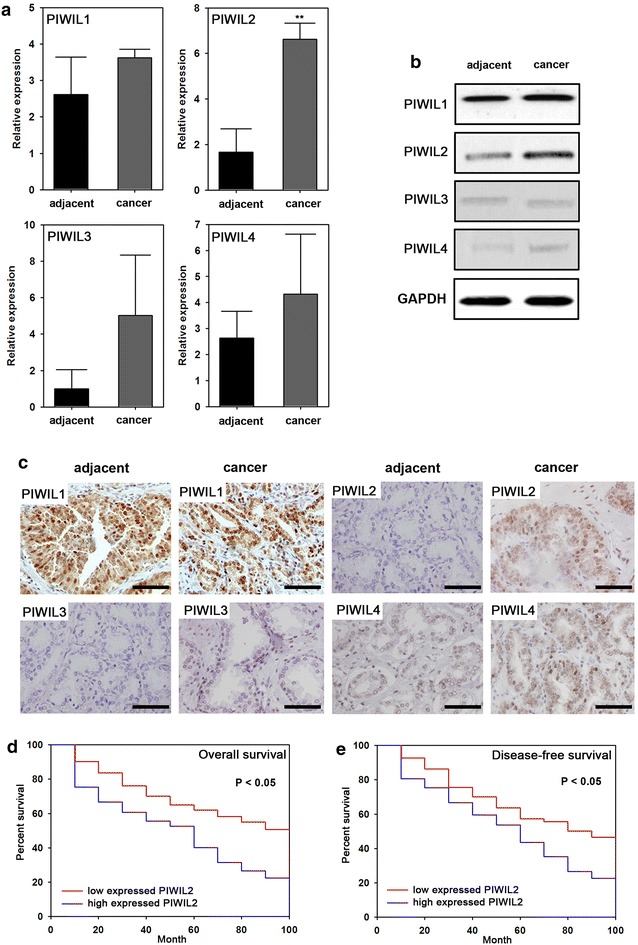
Expression of PIWIL2 in NSCLC and multivariate Cox’s regression analyses of primary NSCLC patients (n = 126). Real-time PCR (**a**), western blot (**b**) and IHC (**c**) analysis of adjacent and NSCLC tissues. **d** Overall survival rates and its correlation with PIWIL2 expression. The cumulative overall survival rate for patients in the PIWIL2-high group was significantly lower than that for patients in the PIWIL2-low group (χ^2^ = 7.54; P = 0.03). **e** Disease-free survival rates and its correlation with PIWIL2 expression. The cumulative disease-free survival rate for patients in the PIWIL2-high group was significantly lower than that for patients in the PIWIL2-low group (χ^2^ = 8.78; P = 0.02). Real-time PCR data are presented as the mean ± SD (n = 30); **P < 0.01; for IHC, *scale bars* = 100 μm. The longest follow-up time was 100 months

To demonstrate the roles of PIWI in NSCLC, real-time PCR, western blot and IHC were employed to assay the expression levels of the four PIWI homologs (PIWIL1, PIWIL2, PIWIL3 and PIWIL4) in NSCLC tissues and adjacent tissues. PIWIL2 expression was significantly higher, both at the mRNA (P < 0.05) (Fig. 1a) and protein levels (Fig. 1b, c), in malignant cancer tissues compared to adjacent tissues. No significant difference was observed in other PIWI genes (PIWIL1, PIWIL3 or PIWIL4) between NSCLC tissues and adjacent tissues (Fig. 1).

### PIWIL2 is associated with poor prognosis in NSCLC

We observed 126 patients for a period of 100 months to determine the relationship between PIWIL2 expression and NSCLC prognosis. Patients were divided into two groups: those that had high levels of PIWIL2 expression (mRNA expression levels higher than 5), and those with low levels of PIWIL2 expression (mRNA expression levels lower than 4). Overall survival and disease-free survival were estimated. We found a significant negative correlation between PIWIL2 and overall survival as well as disease-free survival (Fig. 1d, e).

### PIWIL2 regulates NSCLC cell progression

**Fig. 2:**
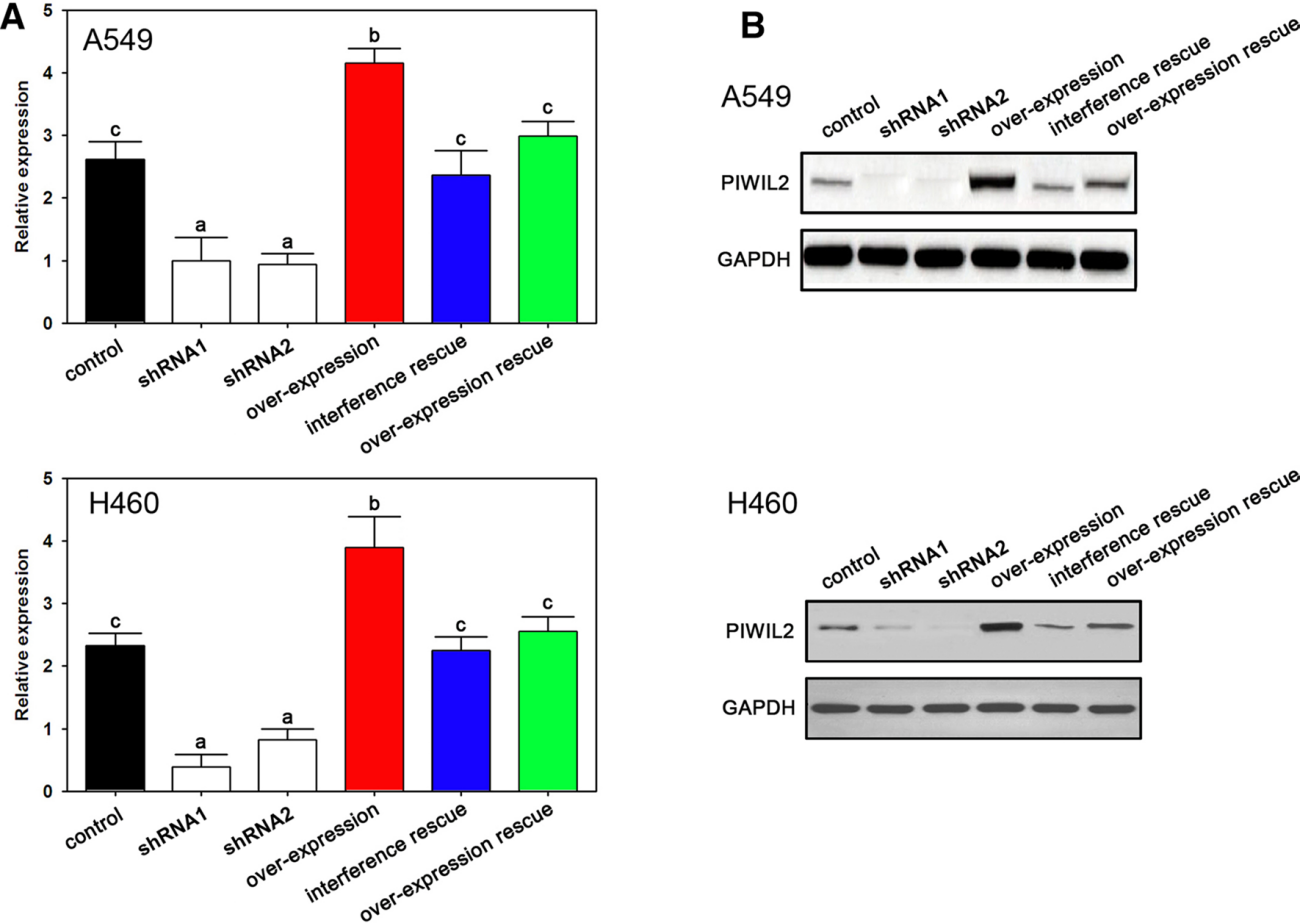
The expression of PIWIL2 in NSCLC cells after transfection. Real-time PCR (**A**) and western blot (**B**) analysis indicated that mRNA and protein expression were down-regulated and up-regulated by interference and over-expression of PIWIL2, respectively

To determine the role of PIWIL2 on NSCLC cells, we used RNA interference and overexpression vectors to control PIWIL2 expression. As expected, the overexpression vectors increased, while the interference assay decreased PIWIL2 levels, as confirmed by real-time PCR (Fig. 2A) and western blot (Fig. 2B). Both shRNA1 and shRNA2 depressed expression of PIWIL2 which confirmed no off target effects in the present experiment (Fig. 2A, B).

**Fig. 3:**
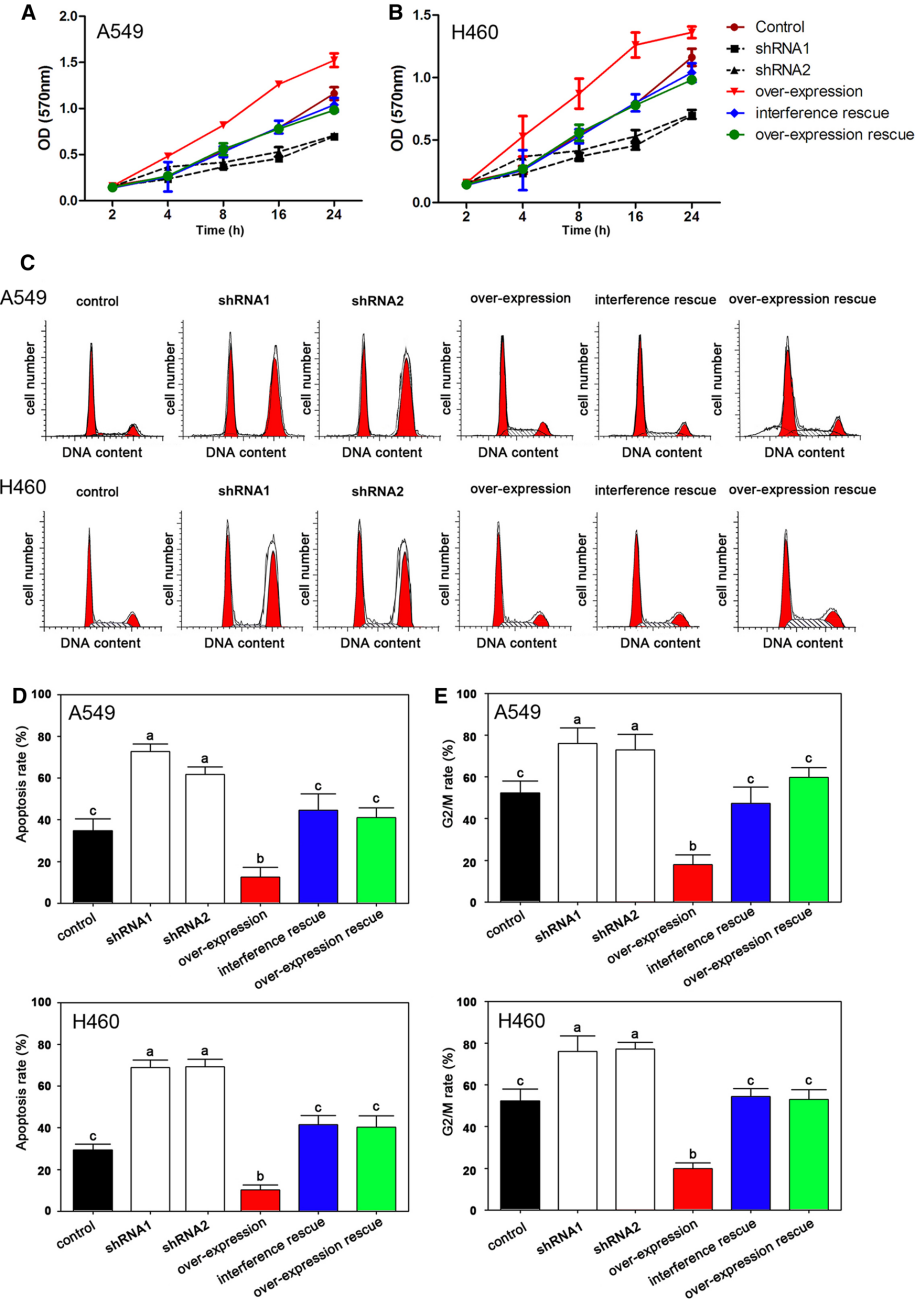
Effects of PIWIL2 on NSCLC cells after transfection. **A** and **B** indicate the cell proliferation after transfection by MTT in A549 and H460. **C** Flow cytometry analysis. **D** Apoptosis rates of PIWIL2 interference and over-expression groups, determined by flow cytometry analysis. **E** G2/M stage arrested rate was increased after repression of PIWIL2. *Different lower case characters* represent significant differences, P < 0.05

The MTT assays indicated that cell proliferation could be depressed by both shRNA1 and shRNA2 interference while overexpression of PIWIL2 induced cell proliferation both in A549 and H460 (Fig. 3A, B). Using flow cytometry, we found that the interference group had higher levels of apoptosis compared with the overexpression group (Fig. 3C, D). Furthermore, in the interference group, more cells arrested at the G2/M stage compared with the other groups (Fig. 3C, E).

### PIWIL2 regulates CDK2 and Cyclin A expression in NSCLC cells

**Fig. 4:**
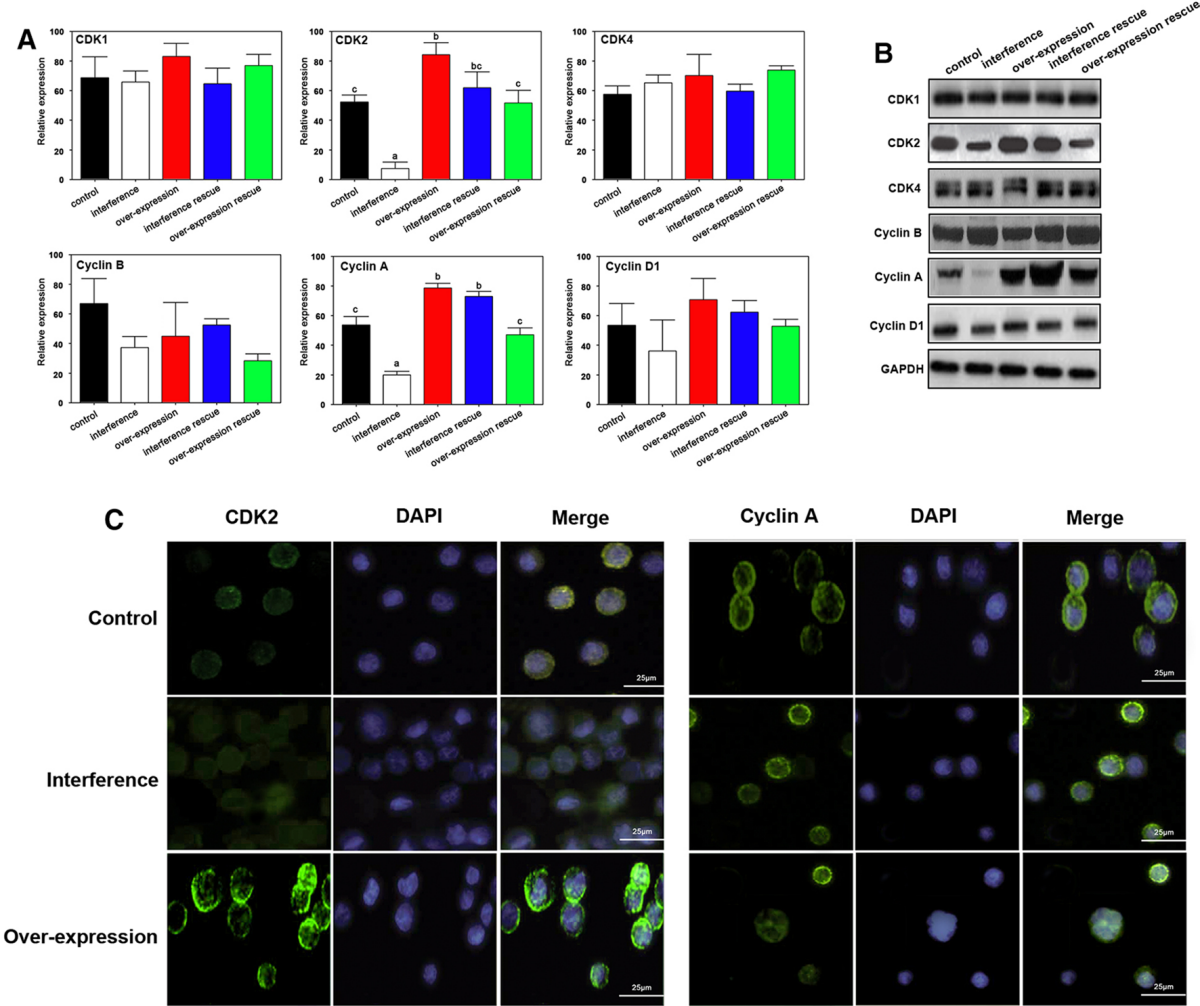
The effects of PIWIL2 on CDK2 and Cyclin A in NSCLC cells. Real-time PCR (**A**) and western blot (**B**) analysis indicated that only CDK2 and Cyclin A is affected by changes in PIWIL2 expression. The *characters on the bars* represent significant differences between groups (P < 0.05). **C** Immunofluorescence analysis of CDK2 and Cyclin A after interference of over-expression of PIWIL2. CDK2 and Cyclin A protein were stained by FITC (*green*). Nuclei were stained by DAPI (*blue*)

We next investigated the mechanism whereby PIWIL2 causes G2/M cell cycle arrest by measuring the expression of Cyclins and CDKs. The interference of PIWIL2 was used shRNA1 which has been proved with no off target effects. The A549 cell line was used to determined the gene expression. The present result indicated that no significant differences were observed for CDK1, CDK4, Cyclin B or Cyclin D at mRNA or at the protein level (Fig. 4A–C). On the contrary, CDK2 and Cyclin A expression were significantly decreased or increased following PIWIL2 interference, or overexpression, respectively (Fig. 4A–C).

### PIWIL2 promotes tumor growth in vivo

**Fig. 5:**
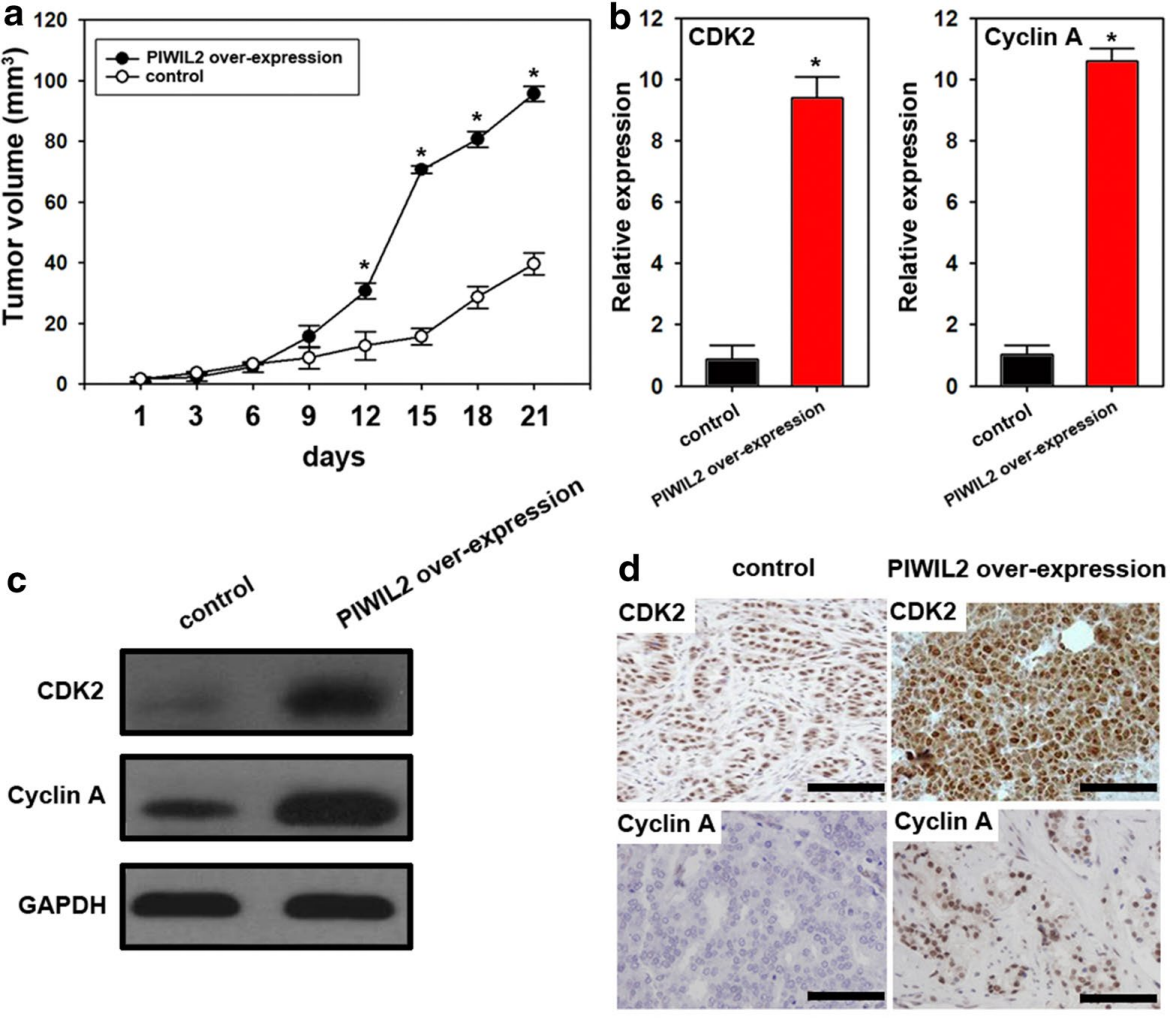
PIWIL2 promotes tumor growth, and CDK2 and Cyclin A expression in nude mice. **a** Injection with A549 cells containing the PIWIL2 over-expression vectors promoted tumor growth in a nude mice model (n = 5). Real-time PCR (**b**), western blot (**c**) and IHC (**d**) analysis indicated that over-expression of PIWIL2 increased CDK2 and Cyclin A expression in tumor tissues. *Asterisk* indicates the significant difference between the groups (P < 0.05). For IHC, *scale bars* = 100 μm

We found that mice treated with PIWIL2-transfected A549 cells had significantly increased tumor volumes compared to controls (Fig. 5a). Moreover, PIWIL2 expression promoted expression of CDK2 and Cyclin A both at mRNA and protein level (Fig. 5b–d), which indicates that PIWIL2 induces CDK2 and Cyclin A expression and promotes tumorigenesis in nude mice.

## Discussion

In this study, we explored PIWI expression in NSCLC as well as its role in tumor progression. By comparing the expression of the four PIWI homologs (PIWIL1, PIWIL2, PIWIL3 and PIWIL4), we showed that only PIWIL2 was highly expressed in malignant NSCLC tissues compared with adjacent tissues. These results suggested that PIWIL2 may play a crucial role in the progression of NSCLC. Moreover, high levels of PIWIL2 expression were associated with decreased overall survival and disease-free survival rates. Similar results have been demonstrated in breast [27] and endometrial cancer [28]. Thus, in cancers, the overexpression of PIWIL2 is key evidence in the malignant and pernicious [30]. However, PIWI expression patterns vary by cancer type. For instance, all PIWI proteins are overexpressed in colon cancer [29], while only PIWIL2 is expressed in breast cancer [26]. Although PIWI genes are potentially useful diagnostic and prognostic biomarkers, the heterogeneous expression patterns between different cancer types is still not well understood. We showed that PIWIL2 is highly expressed in NSCLC and is a potential indicator of NSCLC prognosis.

Once we confirmed the aberrant expression of PIWIL2 in NSCLC, we investigated the potential role of PIWIL2 in NSCLC progression in NSCLC cells using overexpression and interference of PIWIL2. High PIWIL2 expression significantly promoted cell proliferation and activities. Conversely, inhibition of PIWIL2 triggered apoptosis and G2/M cell cycle arrest. It is widely recognized that PIWIL2, as a small RNA binding protein, mediates genome stability and regulates genes expression [32, 33]. The PIWI-piRNA pathway was first illustrated in 2006 [22, 34], however, little is known regarding the PIWI-piRNA pathway in cancer. Although abnormal PIWI expression resulting in poor prognosis has previously been reported, the specific role of PIWI in cancer cells remains unknown. Additionally, PIWIL2 interference triggers apoptosis, which implies a potential therapeutic strategy for NSCLC.

Because PIWIL2 regulates progression of NSCLC cells by controlling the cell cycle, we quantified cell-cycle-related proteins affected by PIWIL2. CDK2 and Cyclin A are key factors that control DNA synthesis and the cell cycle [35]; lack of CDK2 and Cyclin A leads to apoptosis [36]. We showed that PIWIL2 promotes expression of CDK2 and Cyclin A both in vitro and in vivo. In a previous study, PIWIL2 could inhibit TGF-β signaling via Hsp90 and by promoting TβR degradation [37]. Further, PIWIL2 and TGF-β expression are negatively correlated [37]. These findings suggest that PIWIL2 may affect cell proliferation through various means. Our results support previous findings that PIWIL2 promotes cell proliferation.

Over the last decade, evidence has emerged that an epigenetic switch occurs during NSCLC transformation where non-coding RNAs, including miRNAs, piRNAs, and long non-coding RNAs, mediate self-renewal of cancer initiating cells [38]. Lim and colleagues showed that piRNA pathway genes are overexpressed in ovarian cancer [39] and they proposed that PIWIL1 and MAEL inhibit cell invasion. A subsequent study indicated that the RASSF1C-dependent promotion of lung cancer cell proliferation is dependent on IGFBP-5 and PIWIL1, implicating the PIWI-piRNA pathway in tumorigenesis [40]. In vertebrates, abnormal expression of PIWI genes was related to tumorigenesis, sterile and hypogenesis [41–46]. However, until now, the PIWI-piRNA pathway remains poorly understood. In addition to its role in stabilizing the genome, the PIWI-piRNA pathway is likely involved in tumorigenesis. So far, detailed evidence for the role of PIWI proteins in tumorigenesis remains limited. We showed that PIWIL2 interference inhibited NSCLC progression, both in vitro and in vivo, which suggests potential therapeutic value for PIWIL2 in NSCLC.

## Conclusions

In sum, we showed that increased expression of PIWIL2 is associated with worse prognosis of NSCLC and that PIWIL2 interference could inhibit cell proliferation and activities. Furthermore, CDK2 and Cyclin A were regulated by PIWIL2 both in vitro and in vivo. These findings suggest that PIWIL2 participates in the progression of NSCLC via CDK2 and Cyclin A.
